# Effectiveness of oral health promotion interventions for people with type 2 diabetes delivered by non-dental health care professionals: a systematic review: Oral health promotion for people with diabetes by non-dental healthcare professionals

**DOI:** 10.1080/16549716.2022.2075576

**Published:** 2022-08-10

**Authors:** Yuriko Harada, Dilip Prajapati, Prakash Poudel, Biraj Karmacharya, Tomohiko Sugishita, Lal Rawal

**Affiliations:** a Department of International Affairs and Tropical Medicine, Tokyo Women’s Medical University, Tokyo, Japan; b Department of Community and Public Health Dentistry, Dhulikhel Hospital, Kathmandu University School of Medical Sciences, Nepal; c School of Nursing and Midwifery, Western Sydney University, Liverpool, NSW, Australia; d Ingham Institute for Applied Medical Research, Liverpool, NSW, Australia; e Drug Health Services, South Western Sydney Local Health District (SWSLHD), Cabramatta, NSW, Australia; f Department of Community Programs, Dhulikhel Hospital Kathmandu University Hospital, Kathmandu University, Nepal; g School of Health, Medical and Applied Sciences, College of Science and Sustainability, Central Queensland University, Australia; h Physical Activity Research Group, Appleton Institute, Central Queensland University, Australia; i Translational Health Research Institute, Western Sydney University, NSW, Australia

**Keywords:** Oral health, diabetes mellitus, health promotion, health education, primary health care, integration

## Abstract

**Background:**

Oral health problems among people with diabetes mellitus are an emerging public health problem. Despite the rising concerns of oral health and diabetes mellitus comorbidity, there is a lack of dental health care professionals such as dentists, to address this problem, especially at the primary care level in low- and middle-income countries.

**Objective:**

This review systematically synthesizes the current evidence in terms of the involvement of non-dental health care professionals in promoting oral health among people with type 2 diabetes mellitus and assessed the effectiveness of such programs.

**Methods:**

Six electronic databases (CINAHL, Cochrane, Embase, PsycINFO, PubMed, and Scopus) and Google Scholar were systematically searched. The inclusion criteria were: 1) had an intervention promoting oral health; 2) targeted but not limited to people with type 2 diabetes mellitus; 3) intervention led but not limited to by non-dental health care professionals; 4) published in English language between January 2000 and July 2021. This review was registered in PROSPERO (#CRD42021248213).

**Results:**

A total of five studies from four countries (Finland, Thailand, Iran, and the Netherlands) met the inclusion criteria. The interventions included oral health education, a dental care reminder system, and the implementation of oral health care protocols in general practices, all of which were mainly implemented by nurses. All interventions improved clinical outcomes, including decreased probing depth, attachment loss, and plaque index scores, and non-clinical outcomes such as enhanced knowledge, attitudes, and behaviors. Three studies also reported an improvement in diabetes mellitus outcomes.

**Conclusion:**

This review suggests that non-dental health care professionals play a promising role in promoting oral health among people with type 2 diabetes mellitus. Our findings support the potential for integrating oral health promotion programs in primary health care, as such programs could bring better oral health and diabetes mellitus outcomes.

## Background

Diabetes mellitus (DM) is a metabolic disease that causes hyperglycemia, which is classified into four etiology-based categories: type 1 diabetes (T1DM), type 2 diabetes (T2DM), gestational diabetes mellitus (GDM), and other specific types [1]. The global prevalence of DM was 9.3% (463 million people) in 2019, with estimates projecting a prevalence of 10.9% (700 million people) by 2045 [2]. Worldwide, T2DM is the most common condition, accounting for 90% of all people with DM [3]. The rapid increase in T2DM is also occurring in low- and middle-income countries (LMICs) due to aging, unhealthy diets, obesity, and sedentary lifestyles [4, 5,6]. Chronic hyperglycemia leads to complications in various organs, especially the eyes, kidneys, nerves, heart, and blood vessels [1]. Hyperglycemia also results in several oral health problems, primarily periodontal (gum) diseases [7–9]. Periodontal diseases include gingivitis and periodontitis [9,10]. Gingivitis is the mildest form of periodontal disease, a condition that is reversible by improving oral hygiene [9]. On the other hand, periodontitis causes the loss of connective tissue, resorption of alveolar bones, and formation of periodontal pockets [9,10]. Once periodontal pockets fill with bacteria, the condition often becomes irreversible, leading to tooth loss [9,10]. Periodontitis is considered the sixth most prevalent complication of DM [7–9]. According to the global burden of disease study in 2017, 796 million people had periodontal disease, increasing considerably by 50.2% since 1990 [11]. Evidence suggests a bidirectional relationship between T2DM and periodontitis; people with T2DM have a higher risk of periodontitis, which in turn negatively impacts blood glucose control and increases the risk of microvascular and macrovascular complications [7–9]. Furthermore, non-surgical periodontal treatment among people with T2DM can be beneficial for glycemic control [8,12, 13, 14]. Despite people with DM are at greater risk of oral health problems, systematic review showed that the majority of people with T2DM were unaware of the bidirectional relationship between DM and oral health, had limited knowledge of their risk of periodontal diseases, and were less compliant to recommended oral hygiene behaviors ([15]. Moreover, adequate oral health literacy is positively associated with increased frequency of tooth brushing and dental visits, which help to maintain good oral health [16]. Despite the growing burden of DM with periodontal disease comorbidity, there is a lack of dental health care professionals promoting, maintaining, and improving oral health among people with DM, especially in LMICs [17]. Therefore, oral health promotion programs in diabetes care settings could be essential for improving oral health and diabetes outcomes in people with T2DM [18,19].

Studies have emphasized a proactive role of non-dental health care professionals in providing integrated oral health care in primary health care settings [18,19]. Moreover, non communicable diseases such as DM share the common risk factors with oral diseases, so that a involvement of all health care professionals such as physicians, nurses, and community health care workers to promote oral health in people with DM has been emphasized [20]. However, a scoping review conducted in 2016 showed limited evidence on the role of non-dental health care professionals in oral health promotion [21]. Therefore, we conducted a systematic review to update the current evidence base on this area to assess the effectiveness of approaches for promoting oral health for people with T2DM led by non-dental health care professionals. In this study, we considered oral health promotion as non-clinical interventions aimed at improving oral health outcomes.

## Methods

This review was performed using the Preferred Reporting Items for Systematic reviews and Meta-Analyses (PRISMA) guidelines [22]. The protocol for this systematic review was registered on the International Prospective Register of Systematic Reviews (PROSPERO), registration number CRD42021248213.

### Inclusion and exclusion criteria

The retrieved studies were assessed on the following inclusion and exclusion criteria. The inclusion criteria were: 1) reported an intervention promoting oral health; 2) targeted but not limited to people with T2DM; 3) an intervention led but not limited to by non-dental health care professionals; 4) published in English language between January 2000 and July 2021. Our initial literature search showed that there were very limited number of studies available in this area. Therefore, we kept our search strategy and inclusion/ exclusion criteria as broader as possible to allow us determining many relevant studies. Our inclusion criteria were not limited to the people with T2DM and intervention provided only by non-dental health care professionals. Studies were excluded if a clinical intervention was conducted, such as dental treatment or prescribing medication that requires dental health care professionals.

### Search strategy

Literature searches were performed in CINAHL, Cochrane, Embase, PsycINFO, PubMed, and Scopus. Additionally, Google Scholar was searched to identify any other relevant publications. Three primary concepts (oral health, DM, and non-dental health care professionals) were combined using Boolean operators. Medical subject heading (MeSH) terms were also used as appropriate. The keywords regarding oral health were: *oral health, oral hygiene, dental health, dental disease, oral disease, dental problem, periodontal disease, teeth, and gum*. The keywords for DM were: *diabetes mellitus, people with diabetes, patients with diabetes, diabetes patients, glucose intolerance, hyperglycemia, and insulin resistance*. Lastly, the keywords encompassing non-dental health care professionals included: *non-dental professional, nurse, nurse practitioner, doctor, medical doctor, general practitioner, physician, family health doctor, endocrinologist, diabetes educator, dietitian, nutritionist, community health worker, and rural health worker*. Search filters were applied for publication language (English) and date (January 2000 to July 2021). On 11 July 2021, a final search was carried out to include the most recent publications in this systematic review. The complete electronic search strategy used in PubMed is presented in an additional file **(Additional file 1)**. Additionally, the reference lists from all included studies were screened to identify additional possible studies that may meet inclusion criteria. All authors discussed and agreed upon the search strategy.

### Study selection and extraction

**Figure 1.**
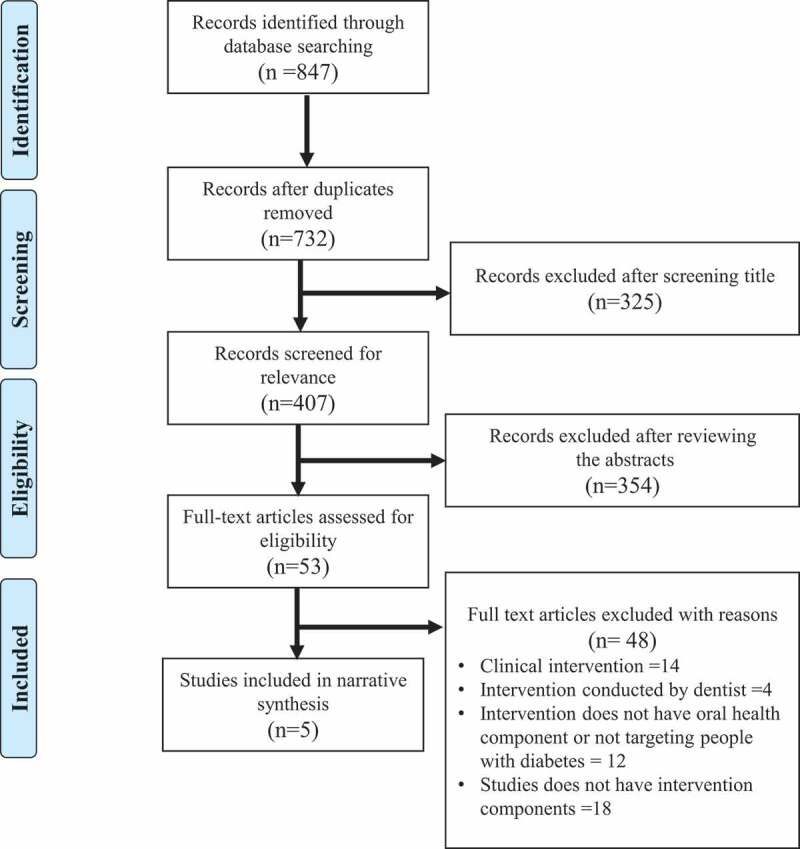
PRISMA flow chart of the study screening process.

The first author (YH) initially identified, imported, and removed duplicates using EndNote bibliographic software. Both YH and DP screened articles by assessing whether the title and abstract met the inclusion or exclusion criteria. Then, YH and DP independently performed the final screening phase by assessing the full texts of the articles retained following the initial screen. Any discrepancies between these two reviewers were resolved through consultation with other authors (PP and LR). A total of five studies met the inclusion criteria and were included in this review (Figure 1).

### Quality assessment and data extraction

Two reviewers (YH and DP) independently assessed the quality of the included studies using the Quality Assessment Tool for Quantitative Studies (QATQS; Table 1) [22]. The QATQS assesses the risk of bias based on six study methodology components; selection bias, study design, presence of confounders, blinding, validity and reliability of data collection methods, and study dropouts and withdrawals [23]. Each component was rated as strong, moderate, or weak, which generated an overall study rating as strong, moderate, or weak. Discrepancies between the two reviewers (YH and DP) were resolved through further discussion and consensus with other authors (PP and LR). In order to allow us including as many studies as possible in this review, the quality assessment of the studies was not the criteria for either to reject or accept the studies in this review. Two studies were rated as weak [24,25], one was moderate [26], and the remaining two were strong [27,28] (Table 1). Variables for data extraction, conducted by the lead author (YH), included: author, year of publication, country, study design, sample size, the mean age of the population, intervention methodology, the professions that led the intervention, intervention duration, dropout rate, oral health outcome indicators, DM and general health outcome indicators, oral health outcomes, and DM and general outcomes. The second author (DP) checked the extracted data for accuracy and completeness.

**Table 1.** Study quality scores.

Author (year)	Selection bias	Study design	Confounders	Blinding	Data collection method	Withdrawals and dropouts	Overall rating
Karikoski A et al. (2003)	Weak	Strong	Weak	Moderate	Weak	Strong	Weak
Saengtipbovorn S et al. (2014)	Moderate	Strong	Strong	Moderate	Strong	Strong	Strong
Saengtipbovorn S et al. (2015)	Moderate	Strong	Strong	Moderate	Strong	Strong	Strong
Malekmahmoodi M et al. (2020)	Weak	Strong	Strong	Moderate	Strong	Strong	Moderate
Verhulst MJ et al. (2021)	Weak	Strong	Strong	Weak	Weak	Moderate	Weak

### Data synthesis

In this review, we identified heterogeneity of the studies in terms of study design, intervention methodology, data analyses and assessment of outcome measures. Since, pooling data to allow us for conducting meta-analyses was not possible, we therefore performed narrative synthesis of data and information provided in each study [29,30].

## Results

### Study location and participant demographics

The location and demographics of the included studies are shown in Table 2. Among the five studies included, two were quasi-experimental studies from Finland [24] and Thailand [27], while three were randomized controlled trials (RCTs) from Thailand [28], Iran [26], and the Netherlands [25]. In total, the five studies had a combined sample size of 1268 people with DM, with the sample size of individual studies ranging from 120 [24,26] to 764 people [25]. The study conducted in Finland included all types of DM, including T2DM [24], but the other studies only included people with T2DM [25–28]. The mean age of the participants ranged from 44.6 years [24] to 67.3 years [25].

**Table 2.** Study location and participant demographics.

Author (year)	Country	Study design	Sample size	Mean age in years old (SD)
Karikoski A et al. (2003)	Finland	Quasi-experimental	120 (T1DM, T2DM, T3DM)	44.6 (13.5)
Saengtipbovorn S et al. (2014)	Thailand	Cluster randomized controlled trial	132 (T2DM)	Intervention group: 63.8 (4.5) Control group: 64.1 (5.5)
Saengtipbovorn S et al. (2015)	Thailand	Quasi-experimental	132 (T2DM)	Intervention group: 63.8 (4.5) Control group: 64.1 (5.5)
Malekmahmoodi M et al. (2020)	Iran	Randomized controlled trial	120 (T2DM)	Intervention group: 53.5 (4.4)Control group: 53.3 (4.5)
Verhulst MJ et al. (2021)	Netherlands	Cluster randomized control trial	764(T2DM)	Intervention group: 64.3 (10.9)Control group: 67.3 (10.3)

### Study Interventions

The interventions are presented in Table 3. The study in Finland was conducted in a diabetes clinic with a control group and three intervention groups: diabetes nurse-letter-reminder group; diabetes nurse-reminder group; letter-reminder group [24]. The diabetes nurse-letter-reminder group received a letter reminding them about dental care as well as receiving a reminder from a diabetes nurse [24]. The diabetes nurse-reminder group was reminded about dental care only by a diabetes nurse, and the letter-reminder group received only a reminder letter [24]. The control group did not receive any reminders [24].

**Table 3.** Study interventions.

Author (year)	Intervention methodology	Profession leading the intervention	Intervention duration	Dropout (%)
Karikoski A et al. (2003)	Diabetes nurse-letter-reminder group Diabetes nurse-reminder groupLetter reminder groupControl group	Diabetes nurses	2 years	4%
Saengtipbovorn S et al. (2014)	**Intervention group**:(Baseline) Lifestyle and oral health education, individual lifestyle counseling, application of self-regulation manual, and individual oral hygiene instruction.(1st and 2nd months) Booster education by viewing an educational video. **Control group**: routine program	Nurse practitioners Dental assistants	3 months	1.5%
Saengtipbovorn S et al. (2015)	**Intervention group**: (Baseline) Lifestyle and oral health education, individual lifestyle counseling, application of a self-regulation manual, and individual oral hygiene instruction.(3rd month) Individual lifestyle counseling and oral hygiene instruction. (1st, 2nd, 4th, and 5th month) Booster education by viewing an educational video **Control group**: routine program	Nurse practitioners Dental assistants	6 months	1.5%
Malekmahmoodi M et al. (2020)	**Intervention group**: Four education sessions **Control group**: Routine care	Not clear^1)^	1 month^2)^	0%
Verhulst MJ et al. (2021)	**Intervention group**: Received oral care protocol including education, referral to the dental care, oral hygiene product **Control group**: Routine care	Genera practitioners Nurses	1 year	29%

The author of this study informed us that the main profession leading the intervention was nurses. The outcome was assessed at three months

Two papers were from the same study conducted in Thailand, one published in 2014 and the other in 2015, however they had different interventions and follow-up lengths [27,28]. The interventions were based on the health belief model, social cognitive theory, and cognitive-behavioral theory [27,28]. At baseline, participants in both studies received a lifestyle and oral health education program, individual counseling, application of self-regulation manual, and oral hygiene instructions [27,28]. The participants also received an educational booster session by viewing a video and nurses’ reminders about educational information [27,28]. The participants of the 2014 study received educational booster sessions in the first and second months, with the study outcome assessed in the third month [27]. On the other hand, participants in the 2015 study received educational boosters in the first, second, fourth, and fifth months, and individual counseling and oral hygiene instruction in the third month, with the study outcome assessed in the sixth month [28]. The interventions of both studies were conducted by nurse practitioners, except for the oral hygiene instructions that were provided by dental assistants [27,28].

The study conducted in Iran had educational interventions based on the health belief model, which emphasizes the awareness, perceived susceptibility, perceived benefit, and perceived self-efficacy and performance of oral hygiene behaviors [26]. The educational intervention was performed over one month, and the study outcome was assessed three months thereafter [26].

Lastly, the study conducted in the Netherlands measured the effectiveness of oral health protocols implemented at the offices of general practitioners (GPs), which included education, encouraging dental visits by providing standardized referral letters, and offering oral hygiene products. Nurses and GPs conducted the intervention, and the study outcome was assessed after one year [25].

### Study outcomes

The outcomes of the interventions for oral health among people with diabetes are presented in Table 4. The study conducted in Finland found that the visible plaque index significantly decreased in all groups, and the calculus index significantly decreased in the diabetes nurse-reminder group and the letter-reminder group [24]. Additionally, mean glycated hemoglobin (HbA1c) decreased from 8.2% (SD 1.3) at baseline to 8.1% (SD 1.4) over the two-year follow-up period, but the reduction was not statistically significant [24].

**Table 4.** Indicators and outcomes of the studies in this review.

Author (year)	Oral health outcomes	Diabetes /general health outcomes	Intervention oral health outcomes	Intervention diabetes/general health outcomes
Karikoski A et al. (2003)	**Clinical outcomes** Visible plaque Presence of calculus CPITN**Non-clinical outcomes** Self-reported increase in tooth brushing, interdental cleaning, and dental visits, Self-reported dental treatment interval and awareness	**Clinical outcomes** HbA1c	A significant decrease in the visible plaque index in all groups, and the calculus index in the diabetes-nurse-reminder group and the letter-reminder group CPITN index codes 3 and 4 increased only in the control group	Mean HbA1c decreased from 8.2% to 8.1%
Saengtipbovorn S et al. (2014)	**Clinical outcomes** Plaque index score Gingival index score Pocket depth Clinical attachment level Percentage of bleeding on probing **Non-clinical outcomes** Knowledge, attitude, behavior, and practice	**Clinical outcomes** HbA1c FPG BMI **Non-clinical outcomes** Knowledge, attitude, behavior, and practice	A significant decrease in plaque index score, gingival index score, pocket depth, clinical attachment level, and percentage of bleeding on probing in the intervention group A significant increase in oral health knowledge, attitude, and some oral health behaviors in the intervention group	A significant decrease in HbA1c from 7.4 to 7.1 and FPG from 143.8 to 129.6 the intervention group No statistically significant difference in BMI between the intervention and control groups A significant increase in diabetes knowledge, attitudes, and some diabetes behaviors in the intervention group
Saengtipbovorn S et al. (2015)	**Clinical outcomes** Probing depth Attachment loss Plaque index score Gingival index score	**Clinical outcomes** HbA1cFPG	A significant decrease in plaque index, gingival index, probing depth, and attachment loss in the intervention group	A significant decrease in HbA1c and FPG in the intervention group
Malekmahmoodi M et al. (2020)	**Non-clinical outcomes** Perceived susceptibility, severity, benefits, barriers, cues to action, and self-efficacy Oral health behaviors	N/A	A significant increase in awareness, perceived susceptibility, benefits, self-efficacy, internal cue to action, and performance in oral and dental hygiene-related behaviors in the intervention group A significant increase in the performance of oral and dental hygiene behaviors in the intervention group	N/A
Verhulst MJ et al. (2021)	**Non-clinical outcomes** Oral-health-related QoL Self-reported oral health complaints	**Non-clinical outcomes** General health-related QoL	Improvements in improved oral health-related QoL in the intervention group Improvements in self-reported oral health did not differ between the groups	No effect on general health-related QoL

BMI = body mass index; CPITN = community periodontal index for treatment Needs; FPG = fasting plasma glucose; HbA1c = glycated hemoglobin; QoL = quality of life

Both studies conducted in Thailand revealed that participants in the intervention group had significantly lower plaque index scores, gingival index scores, pocket depth, clinical attachment levels, and percentage of bleeding on probing compared to the control groups [27,28]. Similarly, the intervention groups of both studies had significantly lower HbA1c and FPG compared to the control groups [27,28]. For example, the 2014 study reported that mean HbA1c decreased from 7.4% (SD 1.2) at baseline to 7.1% (SD 1.0), and that mean FPG decreased from 143.8 mmon/l (SD 38.8) at baseline to 129.6 mmon/l (SD 21.2) after three months follow-up [27].

The study conducted in Iran reported that the awareness, perceived susceptibility, benefits, self-efficacy, internal cue to action, and performance of oral and dental hygiene-related behaviors significantly increased in the intervention group [26]. Specifically, scores for the performance of oral health and dental hygiene behaviors increased from 2.2 (SD 0.7) at baseline to 3.3 (SD 0.5) after three months [26].

Lastly, the study from the Netherlands showed that self-reported oral health-related quality of life (QoL) increased by 35% in the intervention group, compared to 26% in the control group [25]. However, self-reported general health-related QoL were not improved [25].

## Discussion

To the best of our knowledge, this is the first systematic review which summarizes the existing evidence on the effectiveness of approaches for oral health promotion among people with T2DM provided by non-dental healthcare professionals. Overall, five studies showed that oral health interventions such as oral health education, dental care reminder systems, and implementing oral health care protocols in general practices improved oral health status [24–28]. Specifically, clinical outcomes included decreases in probing depth, attachment loss, and plaque index scores, and non-clinical outcomes included enhanced oral health knowledge, attitudes, and behaviors. Additionally, three studies reported improvements in diabetes status, including decreases in HbA1c and FPG [24,27,28]. In terms of quality of evidence, two studies in this review provided strong evidence [27,28].

Nurses were the primary profession providing oral health promotion interventions for people with T2DM [24,28]. While the two studies from Thailand utilized dental assistants for giving oral hygiene instructions, all other interventions were led by nurses [27,28]. The study from Iran did not clearly specify who led the intervention; however, we contacted the author, who informed us that nurses with health education background were the main profession leading the intervention [26]. The study from the Netherlands involved nurses and general practitioners [25]. In previous studies, community health workers, including nurses, have also been effective at improving indicators of diabetes status, such as improved HbA1c and enhanced diabetes knowledge, self-care behaviors, and emotional distress and well-being [31,32]. Furthermore, interventions for diabetes management led by non-physician health care workers, including nurses, were effective in LMICs [33,34]. Our review suggests a promising role for non-dental health care professionals in oral health promotion among people with T2DM.

Previous studies have reported that barriers to oral health promotion by non-dental health care professionals include their limited knowledge and confidence in oral health as they have not received any formal oral health education or training [18,19]. Two studies from Thailand reported that nurses had four days of training on oral health and diabetes care, which included dietary counseling, physical activity, and smoking cessation [27,28]. However, other studies have provided limited information on how non-dental health care professionals received the necessary education to provide oral health interventions. Further studies should address the effectiveness of education strategies to increase the knowledge, confidence, and practical skills of non-dental health care professionals who promote oral health to people with T2DM, especially in LMICs.

This review identified that several intervention methodologies have been implemented, including education, dental care reminder systems, and oral health care protocols in primary health care practices. Despite different methods, all studies reported improvements in oral health status evidenced by clinical and non-clinical outcomes [24–28]. As such, there is potential for integrating oral health promotion programs in diabetes care to improve the oral health status of people living with T2DM. Additionally, studies conducted in Finland and Thailand reported improvements in diabetes status from lifestyle and oral health interventions, and studies from Thailand was rated as high-quality [24,27,28] This supports the bidirectional relationship between oral health and T2DM; improvements in oral health status contribute to better glycemic control [24,27,28]. And this suggests the potential of oral health promotion by non-dental health care professionals to improve glycemic control among people with T2DM in addition to previous literature that supports the benefit of non-surgical periodontal treatment on glycemic control among T2DM people [8,12,,14].Future studies should include larger sample sizes to understand the effectiveness of oral health interventions especially in LMICs, including the cost-effectiveness of such interventions that involve non-dental healthcare professionals, to improve oral health and glycemic status among people living with T2DM.

This review had several limitations. First, we only considered articles published in English, which may have excluded articles published in other languages. Second, the literature search was conducted in only six databases and Google scholar. Therefore, studies available in other databases might have been missed. Third, this review included studies that were conducted among people with T2DM. The results cannot be generalized to people with other types of diabetes, such as T1DM and GDM. Lastly, this review identified only five studies that met the inclusion criteria, and we were unable to conduct meta-analysis due to heterogeneity of the studies as well as lack of adequate data. Despite these limitations, this review has provided an important evidence base for the involvement of non-dental healthcare professionals in the promotion of oral health among people with T2DM.

Our review has important implications for policymakers. Policymakers should develop standardized oral health promotion guidelines and education materials for non-dental healthcare professionals, focusing on the bidirectional relationship between oral health and T2DM. Additionally, efforts should be made to integrate oral health care within primary health care systems, enabling the provision of oral health promotion programs for people with T2DM and those at risk of developing T2DM.

## Conclusion

This systematic review synthesized the current evidence regarding effective approaches for improving oral health among people with T2DM provided by non-dental health care professionals. The findings of this review suggest that non-dental health care professionals can play an important role in promoting oral health for people with T2DM. The interventions provided by non-dental health care professionals effectively improved oral health outcomes, which may be beneficial for glycemic control for people with T2DM. Oral health promotion should be integrated within diabetes care to promote oral health in this high-risk population. Further studies with larger sample sizes are needed to confirm the effectiveness of such interventions, especially in LMICs. Moreover, efforts to develop standardized guidelines and educational materials should be prioritized, focusing on non-dental healthcare professionals providing oral health promotion programs for people with T2DM.

## Paper context

This systematic review explored the effectiveness of oral health promotion among people with type 2 diabetes mellitus led by non-dental health care professionals. Although five studies were identified from a comprehensive database search, all of them reported improvement of oral health status. This support the potential role of non-dental health care professionals and the integration of oral health promotion programs in primary health care for people with type 2 diabetes people.

## References

[1] American Diabetes Association. Diagnosis and classification of diabetes mellitus. Diabetes Care. 2014;37:S81–S90. 24357215 10.2337/dc14-S081

[2] Saeedi P, Petersohn I, Salpea P, et al. Global and regional diabetes prevalence estimates for 2019 and projections for 2030 and 2045: results from the international diabetes federation diabetes atlas, 9th edition. Diabetes Res Clin Pract. 2019;157:107843. 31518657 10.1016/j.diabres.2019.107843

[3] International Diabetes Federation. 2019. IDF Diabetes Atlas. Ninth Edition ed. Brussels; 2019.

[4] Guariguata L, Whiting DR, Hambleton I, et al. Global estimates of diabetes prevalence for 2013 and projections for 2035. Diabetes Res Clin Pract. 2014;103:137–9. 24630390 10.1016/j.diabres.2013.11.002

[5] Petersen PE, Ogawa H. The global burden of periodontal disease: towards integration with chronic disease prevention and control. Periodontol 2000. 2012;60:15–39. 22909104 10.1111/j.1600-0757.2011.00425.x

[6] Zhou B, Lu Y, Hajifathalian K, et al. Worldwide trends 530 in diabetes since 1980: a pooled analysis of 751 population-based studies with 4·4 million participants. The Lancet. 2016;387:1513–1530. 10.1016/S0140-6736(16)00618-8 PMC5081106 27061677

[7] Mealey BL, Oates TW. Diabetes mellitus and periodontal diseases. J Periodontol. 2006;77:1289–1303. 16881798 10.1902/jop.2006.050459

[8] Bascones-Martínez A, González-Febles J, Sanz-Esporrín J. Diabetes and periodontal disease. Review of the literature. Am J Dent. 2014;27:63–67. 25000662

[9] Pihlstrom BL, Michalowicz BS, Johnson NW. Periodontal diseases. Lancet. 2005;366:1809–1820. 16298220 10.1016/S0140-6736(05)67728-8

[10] Williams RC. Periodontal disease. N Engl J Med. 1990;322:373–382. 2405268 10.1056/NEJM199002083220606

[11] James SL, Abate D, Abate KH, et al. Global, regional, and national incidence, prevalence, and years lived with disability for 354 diseases and injuries for 195 countries and territories, 1990–2017: a systematic analysis for the global burden of disease study 2017. Lancet. 2018;392:1789–1858. 30496104 10.1016/S0140-6736(18)32279-7 PMC6227754

[12] Sanz M, Ceriello A, Buysschaert M, et al. Scientific evidence on the links between periodontal diseases and diabetes: consensus report and guidelines of the joint workshop on periodontal diseases and diabetes by the international diabetes federation and the European federation of periodontology. J Clin Periodontol. 2018;45:138–149. 29280174 10.1111/jcpe.12808

[13] Borgnakke WS. IDF Diabetes Atlas: diabetes and oral health – a two-way relationship of clinical importance. Diabetes Res Clin Pract. 2019;157:107839. 31520714 10.1016/j.diabres.2019.107839

[14] Chapple IL, Genco R. Diabetes and periodontal diseases: consensus report of the joint EFP/AAP workshop on periodontitis and systemic diseases. J Periodontol. 2013;84:S106–12. 23631572 10.1902/jop.2013.1340011

[15] Poudel P, Griffiths R, Wong VW, et al. Oral health knowledge, attitudes and care practices of people with diabetes: a systematic review. BMC Public Health. 2018;18:1–12. 10.1186/s12889-018-5485-7 PMC5930945 29716561

[16] Nakre PD, Harikiran AG. Effectiveness of oral health education programs: a systematic review. J Int Soc Prev Community Dent. 2013;3:103. 24778989 10.4103/2231-0762.127810 PMC4000911

[17] Yamalik N, Ensaldo-Carrasco E, Cavalle E, et al. Oral health workforce planning part 2: figures, determinants and trends in a sample of World Dental Federation member countries*. Int Dent J. 2014;64:117–126. 24863646 10.1111/idj.12117 PMC9376428

[18] Poudel P, Griffiths R, Wong VW, et al. Perceptions and practices of general practitioners on providing oral health care to people with diabetes - a qualitative study. BMC Fam Pract. 2020;21:1–11. 32054440 10.1186/s12875-020-1102-9 PMC7020546

[19] Poudel P, Griffiths R, Wong VW, et al. Perceptions and practices of diabetes educators in providing oral health care: a qualitative study. Diabetes Educ. 2018;44:454–464. 30141382 10.1177/0145721718796055

[20] Borgnakke WS, Poudel P. Diabetes and oral health: summary of current scientific evidence for why transdisciplinary collaboration is needed. Frontiers in Dental Medicine. 2021;2:709831.

[21] Poudel P, Griffiths R, Wong VW, et al. Knowledge and practices of diabetes care providers in oral health care and their potential role in oral health promotion: a scoping review. Diabetes Res Clin Pract. 2017;130:266–277. 28662464 10.1016/j.diabres.2017.06.004

[22] Moher D, Shamseer L, Clarke M, et al. Preferred reporting items for systematic review and meta-analysis protocols (PRISMA-P) 2015 statement. Syst Rev. 2015;4:1–9. 25554246 10.1186/2046-4053-4-1 PMC4320440

[23] Quality Assessment Tool for Quantitative Studies. Effective Public Healthcare Panacea Project cited 2021 Sep 5. Available from 2021 Sep 5: https://www.ephpp.ca/quality-assessment-tool-for-quantitative-studies/.

[24] Karikoski A, Ilanne-Parikka P, Murtomaa H. Oral health promotion among adults with diabetes in Finland. Community Dent Oral Epidemiol. 2003;31:447–453. 14986912 10.1046/j.1600-0528.2003.00005.x

[25] Verhulst MJ, Teeuw WJ, Gerdes VE, et al. Implementation of an oral care protocol for primary diabetes care: a pilot cluster-randomized controlled trial. Anna Family Med. 2021;19:197–206. 10.1370/afm.2645 PMC8118494 34180838

[26] Malekmahmoodi M, Shamsi M, Roozbahani N, et al. A randomized controlled trial of an educational intervention to promote oral and dental health of patients with type 2 diabetes mellitus. BMC Public Health. 2020;20:1–9. 32131790 10.1186/s12889-020-8395-4 PMC7057556

[27] Saengtipbovorn S, Taneepanichskul S. Effectiveness of lifestyle change plus dental care (LCDC) program on improving glycemic and periodontal status in the elderly with type 2 diabetes. BMC Oral Health. 2014;14:1–10. 24934646 10.1186/1472-6831-14-72 PMC4069273

[28] Saengtipbovorn S, Taneepanichskul S. Effectiveness of lifestyle change plus dental care program in improving glycemic and periodontal status in aging patients with diabetes: a cluster, randomized, controlled trial. J Periodontol. 2015;86:507–515. 25597411 10.1902/jop.2015.140563

[29] Lisy K, Porritt K. Narrative synthesis: considerations and challenges. JBI Evid Implement. 2016;14(4):201.

[30] Popay J, Roberts H, Sowden A, et al. Guidance on the conduct of narrative synthesis in systematic reviews. A product from the ESRC methods programme (Version I). Lancaster, UK: University of Lancaster; 2006.

[31] Amagyei A, Meal A, Shaw I, et al. Effectiveness of community health worker-led diabetes self-management education on type 2 diabetes patients: a systematic review and meta. Analysis. 2020;1:2.

[32] Trump LJ, Mendenhall TJ. Community health workers in diabetes care: a systematic review of randomized controlled trials. Families, Systems, & Health. 2017;35:320. 10.1037/fsh0000283 28639794

[33] Joshi R, Alim M, Kengne AP, et al. Task shifting for non-communicable disease management in low and middle income countries – a systematic review. PLoS One. 2014;9:e103754. 25121789 10.1371/journal.pone.0103754 PMC4133198

[34] Maria JL, Anand TN, Dona B, et al. Task-sharing interventions for improving control of diabetes in low-income and middle-income countries: a systematic review and meta-analysis. Lancet Glob Health. 2021;9:e170–e80. 33242455 10.1016/S2214-109X(20)30449-6 PMC8279953

